# DPP4 Is a Potential Prognostic Marker of Thyroid Carcinoma and a Target for Immunotherapy

**DOI:** 10.1155/2022/5181386

**Published:** 2022-11-24

**Authors:** Xiaoqian Gao, Yali Le, Chenchen Geng, Zhen Jiang, Guanghui Zhao, Ping Zhang

**Affiliations:** ^1^Department of Ultrasound, Qilu Hospital (Qingdao), Cheeloo College of Medicine, Shandong University, Qingdao 266035, China; ^2^Health Management Center, Qilu Hospital (Qingdao), Cheeloo College of Medicine, Shandong University, Qingdao 266035, China; ^3^Department of Otorhinolaryngology-Head and Neck Surgery, Qilu Hospital (Qingdao), Cheeloo College of Medicine, Shandong University, Qingdao 266035, China; ^4^Medical Laboratory Center, Qilu Hospital (Qingdao), Cheeloo College of Medicine, Shandong University, Qingdao 266035, China

## Abstract

DPP4 (dipeptidyl peptidase 4) is expressed in many cancers, but the relationship between DPP4 and thyroid carcinoma (THCA) is incompletely understood. We aim to explore the expression of DPP4 in THCA and the correlation between DPP4 expression with the prognosis of THCA and antitumor immunity. We systematically analyzed data from The Cancer Genome Atlas (TCGA), Genotype-Tissue Expression (GTEx), and Gene Expression Omnibus (GEO) databases and explored DPP4 expression, its impact on prognosis, and its relationship with antitumor immunity in THCA. Next, we collected 18 pairs of fresh THCA and adjacent paracancerous tissues and performed RT-qPCR to validate the DPP4 mRNA level. Concurrently, immunohistochemistry (IHC) analysis was performed on 12 pairs of paraffin-embedded tissues of medullary thyroid carcinoma (MTC) and paracancerous tissues to validate the DPP4 protein level. Bioinformatics analysis showed that DPP4 mRNA expression in THCA was significantly higher than that in paracancerous tissues (*p* < 0.01). DPP4 was expressed at the highest levels in MTC than in other pathological types. The DPP4 expression level was different between groups with different clinical characteristics. The higher the DPP4 expressed in THCA, the lower the disease-free survival (DFS) was (HR = 1.8, *p*=0.048). DPP4 was significantly correlated with immune cell infiltration and immune response and was positively associated with 21 immune checkpoint genes (ICGs) in THCA (*p* < 0.05). The results of RT-qPCR showed that the relative mRNA expression of DPP4 was significantly upregulated in 18 THCA tissues compared to that in paracancerous tissues (*p*=0.011). IHC results showed that the DPP4 protein level was higher in 12 MTC tissues than in paracancerous tissues (*p*=0.011). In conclusion, DPP4 is a potential prognostic marker of THCA and may become an effective target for immunotherapy.

## 1. Introduction

Thyroid carcinoma (THCA) is the most common malignant tumor of the endocrine system. Primary THCA includes differentiated thyroid carcinoma (DTC), medullary thyroid carcinoma (MTC), and anaplastic thyroid carcinoma (ATC). Patients with DTC have overall better outcomes, and lymphatic metastasis results in poor prognosis in part of the patients [1]. However, MTC, accounting for 2% to 3% of all thyroid malignancies, is less common but more aggressive and shows a poor prognosis [2]. At the time of diagnosis, the incidence of lymphatic metastasis in patients with MTC was up to 75% [3]. Therefore, early diagnosis is critical to advancing outcomes in THCA patients. It is imperative to find more precise prognostic molecular markers.

DPP4, also called CD26 (Cluster of Differentiation 26), is a cell surface glycoprotein. It is located on chromosome 2q24.3, spanning 81.8 kb and containing 26 exons in human [4]. DPP4 is expressed in epithelial cells and endothelial cells in human tissues, and it is also expressed in immune cells such as T cells, B cells, NK cells, and myeloid cells. Its expression level is different in various tumors, and the expression remains unclear in different pathological types of THCA.

Current research indicates that DPP4 functions as a peptidase, regulating chemokine processing, signal transduction, and glucose metabolism. In particular, the essential role of DPP4 in the tumor microenvironment [5] and antitumor immunity [6, 7] is of intense interest. As we know, MTC is an immunoactive tumor described in the literature [8]. Assuming that the expression of DPP4 in THCA is closely related to immune infiltration, it is likely that DPP4 may become a target of immunotherapy. We discussed the correlation between DPP4 and immune infiltration in THCA in this paper. At the same time, DPP4 also plays different roles through different signal pathways in various human solid tumors. DPP4 is an oncogene or tumor suppressor gene in different tumors in humans. Overexpression of DPP4 is associated with poor survival in patients with squamous cell lung cancer [9], hepatocellular carcinoma [10], renal clear cell carcinoma [11], pancreatic carcinoma [12], and colorectal cancer [13]. However, DPP4 is a tumor suppressor in nonsmall cell lung cancer [14], ovarian cancer [15], endometrial cancer [16], and prostatic cancer [17]. Lopez-Campistrous et al. found that high expression of DPP4 was associated with a poor prognosis in THCA patients [18]. It has been proved that DPP4 could bind to some specific molecules, including SNHG7, microRNA95p, and microRNA-152, which seems to affect the growth [19], invasiveness, migration [20], and extrathyroidal extension [21] of THCA. The study [22] shows that DPP4 gene silencing inhibited proliferation and epithelial-mesenchymal transition of PTC cells and enhanced apoptosis by suppressing the MAPK pathway. Thus, DPP4 may be a potential excellent prognostic marker and therapeutic target.

However, the effects of DPP4 on prognosis and immunology in THCA are lacking in systematic research. In our study, we analyzed biological information data from several databases. We focused on the expression level of DPP4, its impact on prognosis, and its relationship with immune checkpoint genes (ICGs), immune microenvironment, and immune response in THCA. To test our hypothesis, we performed real-time quantitative polymerase chain reaction (RT-qPCR) and immunohistochemistry (IHC) to investigate DPP4 expression in THCA and matched adjacent normal thyroid tissues. This study explores the therapeutic potential of harnessing DPP4 as a target to implement THCA immunotherapy, especially MTC, which is more aggressive. We intend to provide new insight for future therapeutic strategies against THCA.

## 2. Materials and Methods

This is an observational study. The raw data from The Cancer Genome Atlas (TCGA) database (https://cancergenome.nih.gov/), Genotype-Tissue Expression (GTEx) database (https://gtexportal.org/home/), and Gene Expression Omnibus (GEO) database (https://www.ncbi.nlm.nih.gov/geo/) were used. At the same time, we collected some fresh THCA nodules and matched adjacent normal thyroid tissues during the procedure for RT-qPCR and IHC. All procedures performed in our study involving human participants were approved by the Ethics Committee of Qilu Hospital of Shandong University (Qingdao) (No. KYLL-KS-2022058). Written informed consent for the scientific use of the biological material was obtained from each patient.

### 2.1. Expression of DPP4 in Human THCA

To ensure the reliability of the result, two databases were used to analyze the expression of DPP4 in human THCA.TIMER (https://cistrome.shinyapps.io/timer/) and GEPIA (https://gepia2.cancer-pku.cn/) were used to analyze the mRNA expression level of DPP4 in human THCA and normal thyroid tissues, respectively. Data from TCGA were analyzed by TIMER using the Wilcoxon test. In the GEPIA database, data from TCGA and GTEx were recomputed by a standard pipeline, making two datasets compatible using multiple analysis methods, including ANOVA and LIMMA. We set the |Log_2_FC| cutoff for 1 and the *p* value cutoff for 0.01 in GEPIA.

### 2.2. Expression of DPP4 in Different Pathological Types of THCA

We selected a THCA dataset via BioGPS (https://biogps.org/). The raw data were from the GEO database. This dataset contains seven different pathological types of THCA. Then, R software with package ggplot2 was used to analyze whether the mRNA expression of DPP4 was different in diverse pathological types of THCA.

### 2.3. Expression of DPP4 in THCA with Different Clinical Features

The online website of UALCAN (https://ualcan.path.uab.edu) was used to analyze whether the expression of DPP4 was different in THCA with different clinical characteristics by the *t*-test. The raw data were from TCGA. Different clinical parameters were set according to the tumor stage, patients with or without lymph node involvement, age of patients, and histological subtypes including classical papillary thyroid carcinoma (CPTC), tall cell variant of papillary thyroid carcinoma (TPTC), follicular variant of papillary thyroid cancer (FPTC), and others. The expression of DPP4 in THCA under different clinical parameters was studied.

### 2.4. Prognostic Effects of DPP4 on THCA

The online website of GEPIA (https://gepia2.cancer-pku.cn/) was used to analyze the relationship between the DPP4 expression level and overall survival (OS) and disease-free survival (DFS) of THCA patients by the log-rank test. The raw data were from TCGA. The median DPP4 expression was applied as a cutoff value to classify groups.

### 2.5. Correlation between DPP4 Expression and ICGs in THCA

We analyzed DPP4 expression and ICGs in THCA via the online website Sangerbox (https://sangerbox.com). The website calculated Pearson's correlation coefficients between ICGs and immune infiltration scores in THCA using the corr.test function of R software package psych (version 2.1.6). In the following 2.6 and 2.7, the statistical methods applicated in the Sangerbox website were the same.

### 2.6. Correlation between DPP4 and Immune Infiltration Cells in THCA

We used the online website Sangerbox (data sources: TCGA databases) to analyze the correlation between DPP4 expression and immune infiltration cells in THCA immune microenvironment. Next, TIMER was used to verify the previous result (data source: TCGA databases).

### 2.7. Scores of Immune Infiltration

The ESTIMATE score, stromal score, and immune score were calculated by the ESTIMATE algorithm using the Sangerbox website. The raw data were from TCGA and GTEx.

### 2.8. Coexpression Network and the Characteristic of Immune Response of DPP4

We explored the coexpression network of DPP4 by using the LinkedOmics website to verify the potential function of DPP4 in THCA. Next, Gene Set Enrichment Analysis (GSEA) was performed. Gene ontology (GO) enrichment analysis (biological process) and Kyoto encyclopedia of genes and genomes (KEGG) pathway analysis were performed.

### 2.9. Expression Level of DPP4 mRNA in THCA Detected by RT-qPCR

A total of 18 pairs of THCA nodules and matched adjacent normal thyroid tissues were collected during the initial surgery, including 14 pairs of papillary thyroid carcinoma (PTC), two pairs of MTC, one pair of follicular thyroid carcinoma (FTC), and one pair of ATC. Samples after surgical resection were immediately put into RNAlater, snap-frozen, and stored at −80°C before extraction. All samples were confirmed by postoperative histopathological examination. We performed RT-qPCR to investigate DPP4 mRNA expression in 18 pairs of matched tissues. The obtained data were analyzed by R software with package ggplot2, and the Mann–Whitney *U* test was used as the statistical method.

### 2.10. Expression Level of DPP4 Proteins in MTC Detected by IHC

The expression level of DPP4 proteins in MTC and matched adjacent normal thyroid tissues from 12 patients was evaluated by IHC. Tissues were processed for standard paraffin embedding. Then, 5 *μ*m tissue sections were collected on glass slides. Performed by a pathologist with ten years of experience in IHC operation, the expression of DPP4 protein was detected by standardized procedures, including dewaxing, antigen retrieval, DPP4 antigen-antibody binding reaction, diaminobenzidine (DAB) staining, hematoxylin staining, and so on. All the IHC images were quantified by Image-pro Plus (6.0 version). We calculated the staining area and integrated optical density (IOD) of each image, and we took the ratio IOD/area which was the average optical density (AOD). The finally obtained paired data were analyzed by the *t*-test using R software package ggplot2.

## 3. Results

### 3.1. mRNA Expression Level of DPP4 in THCA Was Higher than that in Normal Thyroid Tissues

**Figure 1:**
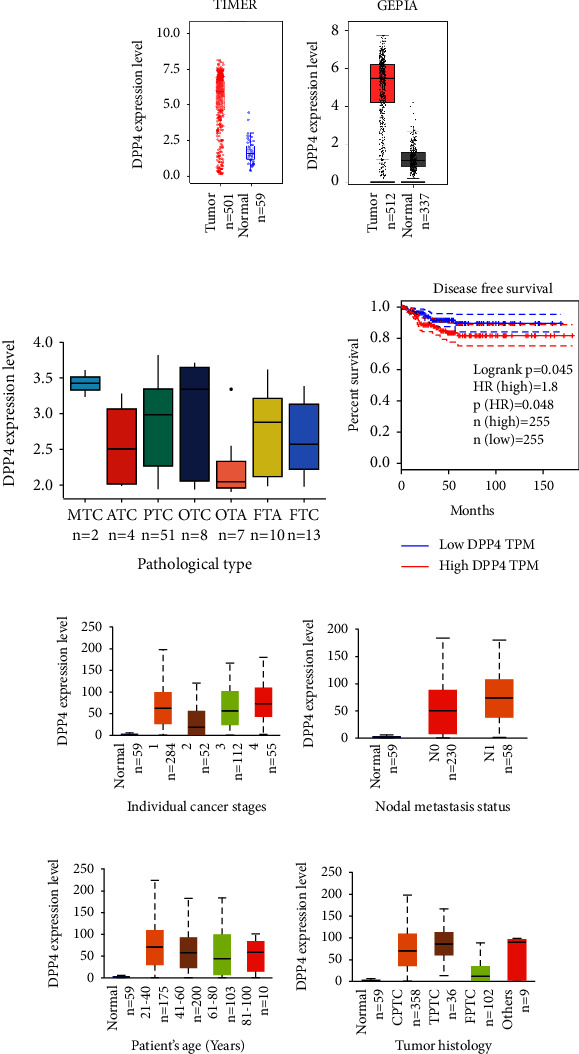
The differential expression of DPP4 and its effect on prognosis in THCA. (a) The analysis result from TIMER: the mRNA expression level of DPP4 in THCA is statistically higher than that in normal thyroid tissues (*p* < 0.001). (b) The result from GEPIA is consistent with that of A (*p* < 0.01). (c) Expression of DPP4 at the mRNA level in 7 different pathological types of thyroid nodules (*p* > 0.05). (d) DPP4 expression significantly affects DFS of THCA patients (*p*=0.048). (e) DPP4 expression is different in patients with different clinical stages of THCA, Stage 2 vs. Stage 3 (*p* < 0.01), and Stage 2 vs. Stage 4 (*p* < 0.001). (f) DPP4 expression is different in THCA patients with different lymph node metastatic status, N0 vs N1 (*p* < 0.05). (g) DPP4 expression is different in different age groups of THCA patients, Age (21–40 Years) *vs.* Age (41–60 Years) (*p* < 0.05), and Age (21–40 Years) *vs.* Age (61–80 Years) (*p* < 0.01). (h) There are differences in DPP4 expression among THCA patients with different histological subtypes, CPTC *vs.* TPTC (*p* < 0.05), CPTC *vs.* FPTC (*p* < 0.001), and TPTC *vs.* FPTC (*p* < 0.001).

The data from TCGA were analyzed by TIMER, and the result showed that the mRNA expression level of DPP4 in THCA was higher than that in normal thyroid tissues statistically (Figure 1(a)) (*p* < 0.001). Then, GEPIA was used to integrate the data from TCGA and GTEx for analysis; the latter contained more normal thyroid tissues, which makes up for the deficiency of normal tissue samples in TCGA. Both the results were consistent (Figure 1(b)) (*p* < 0.01).

### 3.2. DPP4 mRNA Expression Was Higher in MTC

A dataset named human thyroid adenomas, carcinomas, and normals was analyzed. This dataset contained 99 human samples of various thyroid nodules and normals, which consisted of 4 normals, 10 follicular thyroid adenomas (FTA), 13 FTC, 7 oncocytic thyroid adenomas (OTA), 8 oncocytic thyroid carcinomas (OTC), 51 PTC, 4 ATC, and 2 MTC. After removing 4 normals, we analyzed the rest of the seven kinds of thyroid nodules and drew a box plot (Figure 1(c)). The result showed that the mRNA level of DPP4 was lowest in benign nodules of OTA and highest in MTC nodules, although the difference was not significant (*p* > 0.05). We speculate that it may be related to the small sample size.

### 3.3. DPP4 Expression Level Varied in THCA with Different Clinical Features

Patients were divided into groups according to different clinical parameters. DPP4 expressed higher in advanced tumor stage groups, Stage 2 *vs.* Stage 3 (*p* < 0.01) and Stage 2 *vs.* Stage 4 (*p* < 0.001) (Figure 1(e)). The expression of DPP4 in the group with lymph node metastasis was higher than that in the group without lymph node metastasis, N0 *vs.* N1 (*p* < 0.05) (Figure 1(f)). DPP4 expressed higher in younger groups, Age (21–40 Years) *vs.* Age (41–60 Years) (*p* < 0.05), and Age (21–40 Years) *vs.* Age (61–80 Years) (*p* < 0.01) (Figure 1(g)). Among the three histological subtypes, there were statistical differences, respectively, CPTC *vs.* TPTC (*p* < 0.05), CPTC *vs.* FPTC (*p* < 0.001), and TPTC *vs.* FPTC (*p* < 0.001) (Figure 1(h)).

### 3.4. DPP4 Influences the Prognosis of THCA

Through the analysis of GEPIA, there was no significant difference in OS between high and low expression of DPP4 groups (*p*=0.23). Although the effect of DPP4 on OS was not significant (Supplementary Figure 1), it significantly affected DFS (*p*=0.048) (Figure 1(d)). The higher the DPP4 expressed in THCA, the lower the DFS was (HR = 1.8, *p*=0.048) (Figure 1(d)).

### 3.5. DPP4 Expression Was Correlated with ICGs in THCA

**Figure 2:**
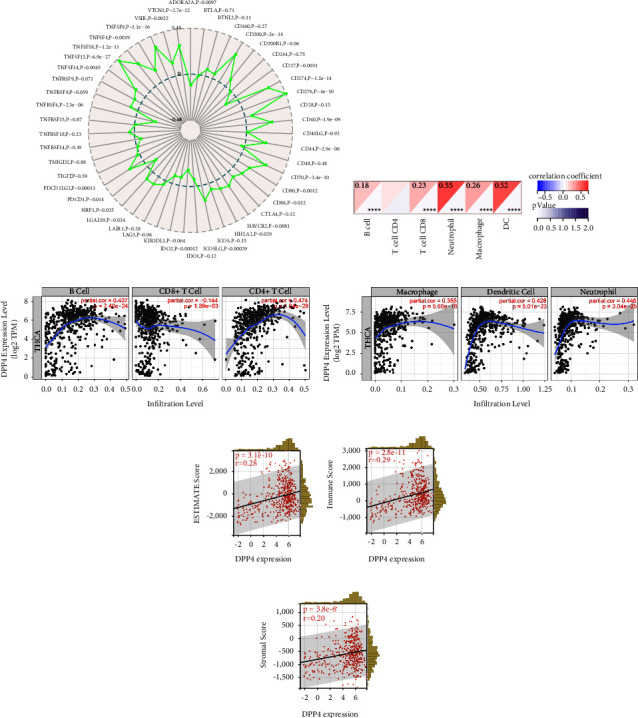
The relationship between DPP4 and THCA tumor immunity. (a) The correlation between the expression of 47 ICGs and DPP4 in THCA is analyzed. Among them, 21 ICGs were positively correlated with DPP4 and 5 were negatively correlated. (b) The results from Sangerbox show that the expression of DPP4 is positively correlated with B cells, CD8+T cells, neutrophils, macrophages, and DCs in THCA (^*∗∗∗∗*^*p* < 0.001). (c, d) The results from TIMER verify that the expression of DPP4 is positively correlated with B cells, CD4+T cells, neutrophils, macrophages, and DCs in THCA (*p* < 0.001). The scatterplots show partial Spearman's rho value. (e) DPP4 expression is significantly correlated with the ESTIMATE score in THCA (*p* < 0.001). (f) DPP4 expression is significantly correlated with the immune score in THCA (*p* < 0.001). (g) DPP4 expression is significantly correlated with the stromal score in THCA (*p* < 0.001).

THCA samples (*n* = 510) from TCGA databases were analyzed. The results of the Sangerbox website analysis showed that among the 47 ICGs, 26 of them were significantly correlated with the expression of DPP4 (*p* < 0.05). Among them, 21 had a positive correlation and 5 had a negative correlation with DPP4 expression (Figure 2(a)). When DPP4 is highly expressed, these ICGs may be regulated in different signaling pathways, resulting in the simultaneous high expression of 21 ICGs. CD276 was one of the positively related ICGs (*p* < 0.001). Previous investigation indicated that a higher CD276 expression level was associated with a high risk of mortality [23]. This demonstrated that THCA with highly expressed DPP4 was probably an immunoactive tumor that could benefit from immunotherapy. DPP4 was a potentially effective target for immunotherapy.

### 3.6. DPP4 Was Correlated with Immune Cell Infiltration of THCA

For THCA samples from TCGA (*n* = 503), we used the Sangerbox website for immunocytic analysis and evaluated each patient's immune cell infiltration score according to the expression of DPP4. The results showed that the expression of DPP4 was positively correlated with B cells, CD8+T cells, neutrophils, macrophages, and dendritic cells (DCs) (*p* < 0.001) (Figure 2(b)). It was verified by TIMER that the expression of DPP4 was positively correlated with B cells, CD4+T cells, neutrophils, macrophages, and DCs (*p* < 0.001) (Figures 2(c) and 2(d)).

### 3.7. DPP4 Expression Level Was Significantly Correlated with Immune Infiltration in THCA

In THCA cases (*n* = 503), the expression of DPP4 was positively correlated with the ESTIMATE score, immune score, and stromal score (*p* < 0.001) (Figures 2(e)–2(g)). The higher the DPP4 expressed, the higher the previously mentioned scores were, suggesting the higher the immune infiltration level.

### 3.8. Coexpression Network of DPP4 Related to Immune Response

The above results suggested that DPP4 was closely related to the prognosis and immunity of THCA. Analysis via LinkedOmics showed the coexpression network of DPP4. The red dots were genes positively related to the expression of DPP4 and the green dots negatively (Supplementary Figure 2). The heat plots (Supplementary Figures 3 and 4) showed the top 50 genes positively and negatively correlated with DPP4 expression, respectively.

**Figure 3:**
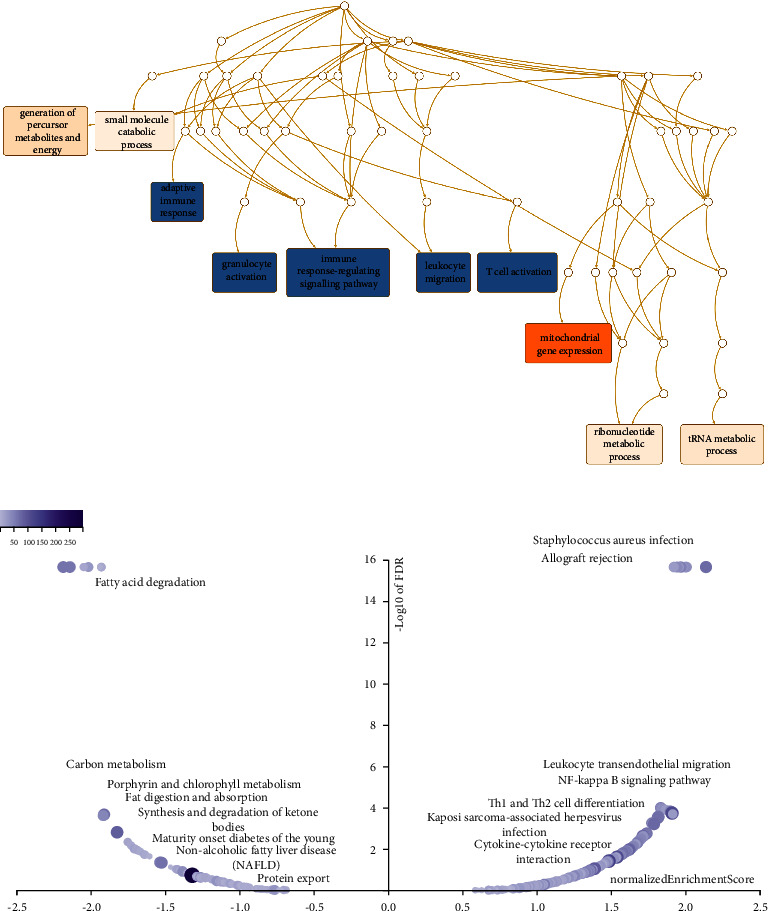
DPP4 enrichment analysis in THCA. (a) GO analysis (biology process) shows that DPP4 and its coexpressed genes are mainly involved in adaptive immune response, granulocyte activation, the immune response-regulating signaling pathway, leukocyte migration, and T cell activation. (b) KEGG pathway analysis indicates that DPP4 coexpression genes enrich in leukocyte transendothelial migration, the NF-kappa B signaling pathway, Th1 and Th2 cell differentiation, and cytokine-cytokine receptor interaction.

Next, through GO analysis (biology process), we found out that DPP4 and its coexpressed genes were mainly involved in adaptive immune response, granulocyte activation, immune response-regulating signaling pathway, leukocyte migration, and T cell activation (Figure 3(a)). The results of KEGG pathway analysis indicated that DPP4 coexpression genes were enriched in leukocyte transendothelial migration, NF-kappa B signaling pathway, Th1 and Th2 cell differentiation, and cytokine-cytokine receptor interaction (Figure 3(b)).

All these results suggested that the expression of DPP4 may play an essential role in THCA by regulating the immune response of TME.

### 3.9. Expression of DPP4 in Fresh THCA Tissues Was Higher than that in Matched Adjacent Normal Thyroid Tissues at the mRNA Level

**Figure 4:**
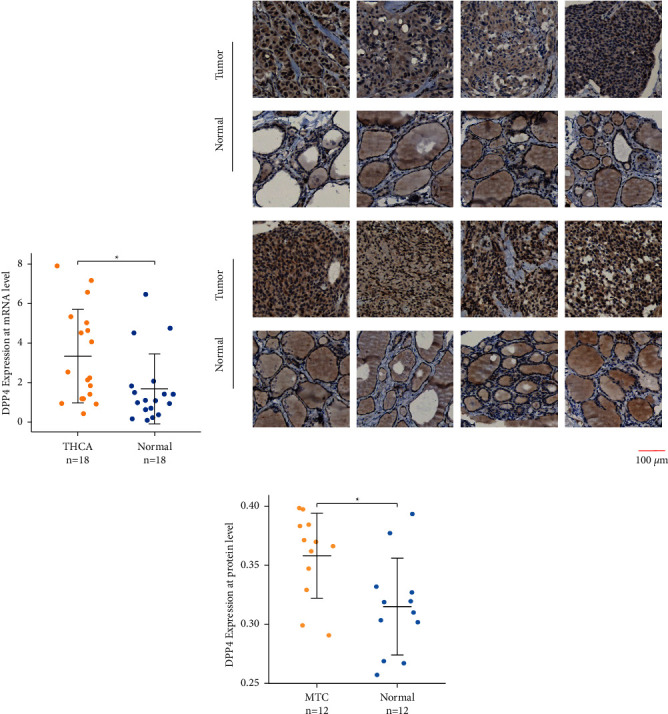
Results of RT-qPCR and IHC. (a) Result of RT-qPCR shows that expression of DPP4 in fresh THCA tissues is higher than that in MANT at the mRNA level (^*∗*^*p* < 0.05). (b) The IHC of 8 pairs of MTC and paracancerous tissues shows that the target protein DPP4 is mainly distributed in the cytoplasm of cancer cells and is stained brown by DAB, while the thyroid follicular epithelium is slightly stained. (c) Quantitative analysis shows that the staining degree in MTC is higher than that in paracancerous tissues (^*∗*^*p* < 0.05).

The analysis results of the RT-qPCR indicated that the relative mRNA expression of DPP4 is significantly upregulated in THCA compared to matched adjacent normal thyroid tissues in 18 pairs of matched tissues (*p*=0.011) (Figure 4(a)).

### 3.10. Expression of DPP4 in MTC Was Higher than that in Matched Adjacent Normal Thyroid Tissues at the Protein Level

The identified proteins were probed by IHC, being stained brown by DAB. It represented that the brown was mainly distributed in the cytoplasm of MTC cancer cells, while the thyroid follicular epithelium of paracancerous tissues was only slightly stained (Figure 4(b) and Supplementary Figure 5). The results of the quantitative analysis showed that the staining degree of the target protein in the MTC group was significantly higher than that in the matched adjacent normal thyroid tissue group (*p*=0.011) (Table 1, Figure 4(c)), which meant expression of DPP4 in MTC was higher than that in matched adjacent normal thyroid tissues at the protein level.

## 4. Discussion

Several types of THCA have a poor prognosis, usually accompanied by extrathyroidal extension and lymph node metastasis. Currently, the underlying molecular mechanisms of THCA are not studied comprehensively and systematically. MTC, which originates from thyroid gland C cells, is characterized by a relatively high degree of malignancy and occurs in two forms, hereditary MTC (hMTC) or sporadic MTC (sMTC). Activation of the RET proto-oncogene by germline mutations was considered the etiology of approximately 25% of MTC cases [24]. About 80% of sMTC cases existed with somatic mutations, containing mainly RET and a few RAS mutations [25]. Qu et al. [26] also attempted to divide sMTC into two molecular subtypes according to FAT1/FAT4 to predict its invasiveness.

Our systematic analysis of bulk clinical samples from TCGA, GTEx, and GEO revealed the relationship between DPP4 and THCA. The mRNA expression level of DPP4 in THCA was higher than that in normal thyroid tissues (*p* < 0.001), which meant DPP4 was a differential gene of THCA. At the same time, the RT-qPCR results of 18 pairs of THCA and matched adjacent normal thyroid tissues also confirmed significant differences in the mRNA expression level of DPP4 in paired tissues (*p*=0.011). Further analysis showed a clear trend that, among the seven types of thyroid nodules, DPP4 expressed higher in malignant nodules than in benign nodules, especially the most elevated one in MTC nodules, although the difference was not significant (*p* > 0.05). This was reliably demonstrated by IHC. In 12 pairs of MTC and matched adjacent normal thyroid tissues, the expression of DPP4 was significantly higher in MTC at the protein level (*p*=0.011). It was indicated that DPP4 played an essential role in THCA, especially in MTC, which may play a carcinogenic role.

In addition, there were statistical differences in the expression of DPP4 in THCA with different clinical features. Notably, DPP4 expressed higher in advanced tumor stages than in the early stage (*p* < 0.05). Meanwhile, DPP4 expressed higher in patients with lymph node involvement than in patients without. Both results suggested that increased expression of DPP4 is related to the aggression of THCA. We also noticed that DPP4 expression was higher in the younger group. Sugino et al. [27] disclosed that the specific characteristics of pediatric and adolescent DTC were more frequent occurrences of distant metastasis compared with adult DTC. This also indirectly proved a specific relationship between DPP4 and the invasiveness of DTC.

Furthermore, we explored the prognostic effect of DPP4 on THCA patients. Poor prognosis was connected to a higher DPP4 expression level. However, Abooshahab et al. [28] showed that the fluctuation of the serum DPP4 level did not play an essential role in the prognosis of patients with MTC; therefore, a higher DPP4 level could not be regarded as a risk factor for MTC. So, whether DPP4 has prognostic value for MTC or not is controversial.

Previous studies have shown that ICGs are related to immune infiltration and prognosis. ICGs can activate the immune checkpoint pathway and prevent tumor cells from being recognized by the immune system, thus inhibiting immune response [29, 30]. The successful application of ICG-inhibitors (ICI) such as anti-CTLA-4 and anti-PD-1/PD-L1 in melanoma, lung cancer, and other cancers makes immunotherapy a feasible treatment for patients with advanced cancer [31]. A study of nonsmall cell lung cancer confirmed that patients with low expression of ICGs had a significantly prolonged survival period [32], which meant that tumor patients would benefit from immunotherapy of ICI. Our results showed that DPP4 was positively correlated with ICGs as a whole in THCA, which provided evidence for the effectiveness of ICI in patients with THCA and laid a foundation for further research. Patients with MTC, an immunoactive tumor with poor prognosis, commonly suffered from lymphatic metastasis and distant metastasis, including osseous and pulmonary metastasis. For these patients who were not suitable for surgery, DPP4 might be a potential target for immunotherapy.

There was a positive correlation between DPP4 and the infiltration of multiple immune cells in the TME of THCA. Tumor-infiltrating lymphocytes (TIL) were proven to be associated with the tumor phenotype, the prognosis of patients [33], and the effect of immunotherapy [34]. Our result represented that there was a strong correlation between DPP4 and TIL. A positive association was observed between DPP4 and B cell, CD8+ T cell, neutrophil, macrophage, and DC. CD8+ T cell infiltration was an independent risk factor of PTC recurrence [35]. Meanwhile, a high density of CD8+ T cells was observed in ATC with a higher degree of malignancy [36].

Besides tumor cells, stromal cells and immune cells are important components in tumors, playing an important role in tumor biology. “Estimation of STromal and Immune cells in MAlignant Tumours using Expression data” (ESTIMATE) is an algorithm including the estimate score, stromal score, and immune score used to calculate the ratio of stromal and immune cells in tumors [37]. Its result indicated a positive relationship between DPP4 expression and immune infiltration. The previous three scores are closely related to TME, various immune-related biological processes, and various immune cells. The scores represent tumor purity. In many malignant tumors such as primary gastric cancer [38] and uveal melanoma [39], the higher the score, the higher the purity of tumor cells and the worse the prognosis. Meanwhile, a higher immune score represents the more robust immune activity of the tumor so that patients may benefit from immunotherapy [40].

The results of GSEA showed that DPP4 was closely related to a variety of immune responses, such as adaptive immune response, granulocyte activation, immune response-regulating signaling pathway, leukocyte migration, and T cell activation. This indicated that DPP4 might play an essential role in THCA by regulating the immune response of TME.

In summary, the previously mentioned findings indicate that DPP4 plays a significant role in the immune process of THCA patients. THCA with high expression of DPP4 may be an immune-active tumor and is suitable for immunotherapy. DPP4 may become an effective target for immunotherapy.

However, this study has some limitations. Our paper analyzed biological information from different databases through different websites. The analysis results between these websites or databases may have varying deviations or even wholly different results. It is not clear how DPP4 is involved in the development of THCA and how it affects the prognosis. More in vitro and in vivo experiments are needed to confirm the results. In addition, whether there is a close relationship between DPP4 and the occurrence and development of MTC needs further experimental research to provide more convincing evidence.

## 5. Conclusion

In this paper, through bioinformatics analysis and experimental verification of mRNA and protein levels, it is found that DPP4 may be a carcinogenic gene of THCA, which is related to the prognosis and antitumor immunity of THCA patients. DPP4 may become an important prognostic marker and immunotherapy target of THCA.

## Figures and Tables

**Table 1:** Staining degree of target protein in paired tissues.

Patients groups	AOD (MTC group)	AOD (MANT group)
1	0.398423776	0.319521728
2	0.371266983	0.268768007
3	0.329106072	0.309980677
4	0.397353359	0.301731287
5	0.366147579	0.326989566
6	0.369798632	0.318775201
7	0.384363123	0.393388335
8	0.383202165	0.377171277
9	0.361845673	0.25711852
10	0.290651666	0.303414949
11	0.29909951	0.26701531
12	0.347160157	0.33187071

AOD: average optical density; MTC: medullary thyroid carcinoma; MANT: matched adjacent normal tissues.

## Data Availability

The data used to support the findings of this study are included within the article and the supplementary information files. The raw data of qPCR and IHC in this study are available from the corresponding author upon request.

## Supplementary Materials

Supplementary Materials See Supplementary Figures 1–5 in the Supplementary Materials for comprehensive image analysis.
