# Supplementary material for: DPP4 Is a Potential Prognostic Marker of Thyroid Carcinoma and a Target for Immunotherapy
Source: Int J Endocrinol. 2022 Nov 24;2022:5181386. doi: 10.1155/2022/5181386 (PMC9715318; doi:10.1155/2022/5181386)
Supplement: Supplementary Materials — See Supplementary Figures 1–5 in the Supplementary Materials for comprehensive image analysis. [file 5181386.f1.zip › supplementary figure legends (1).docx]

**Supplementary figure legends**

**Supplementary figure1.** DPP4 expression influences on OS.

There’s no significance(p>0.05).

**Supplementary figure 2.** Co-expression network of DPP4.

Analysis via Linkedomics shows the co-expression network of DPP4. The red dots are genes positively related to the expression of DPP4 and the green dots negatively.

**Supplementary figure 3.** Top 50 genes positively correlated with DPP4.

**Supplementary figure 4.** Top 50 genes negatively correlated with DPP4.

**Supplementary figure 5.** The IHC of additional 4 pairs of MTC and paracancerous tissues shows that the target protein DPP4 is mainly distributed in the cytoplasm of cancer cells and is stained brown by DAB. The thyroid follicular epithelium is slightly stained.
