# Supplementary material for: DPP4 Is a Potential Prognostic Marker of Thyroid Carcinoma and a Target for Immunotherapy
Source: Int J Endocrinol. 2022 Nov 24;2022:5181386. doi: 10.1155/2022/5181386 (PMC9715318; doi:10.1155/2022/5181386)
Supplement: Supplementary Materials — See Supplementary Figures 1–5 in the Supplementary Materials for comprehensive image analysis. [file 5181386.f1.zip › supplementary figures and legends (1).pdf]

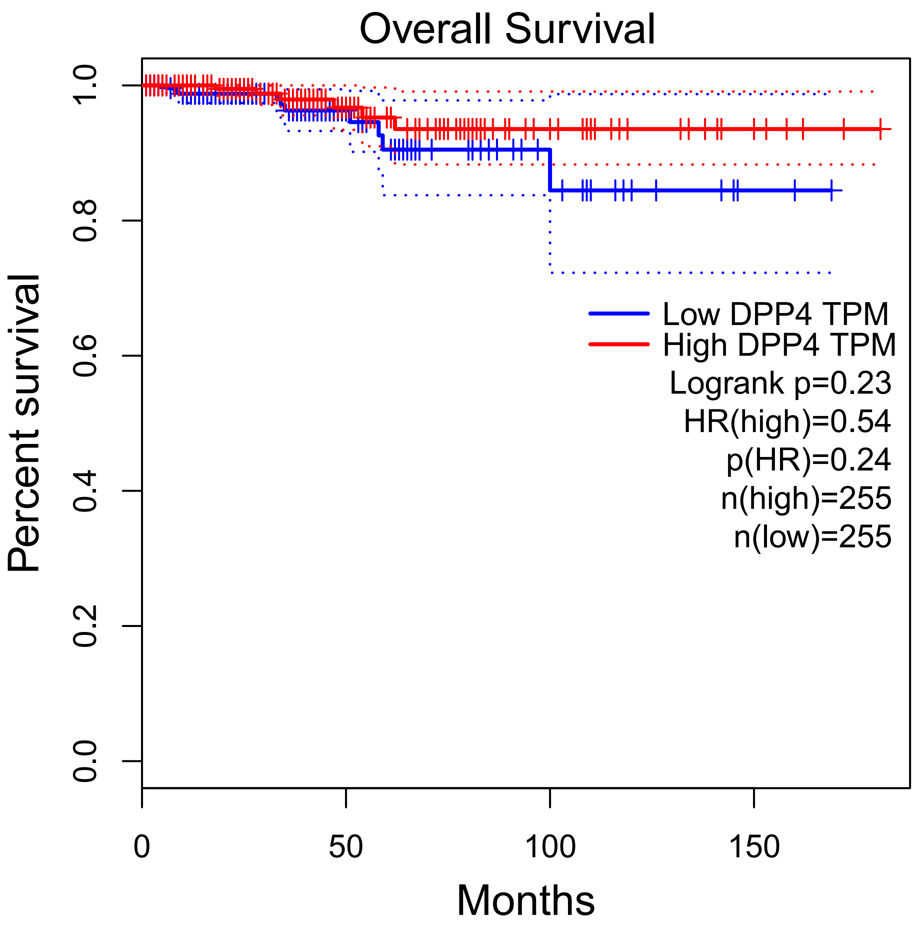

Overall Survival
Percent survival
0.0
0.2
0.4
0.6
0.8
1.0
0
50
100
150
Months
Low DPP4 TPM
High DPP4 TPM
Logrank p=0.23
HR(high)=0.54
p(HR)=0.24
n(high)=255
n(low)=255

Supplementary figure1. DPP4 expression influences on OS.

There’s no significance($p>0.05$).

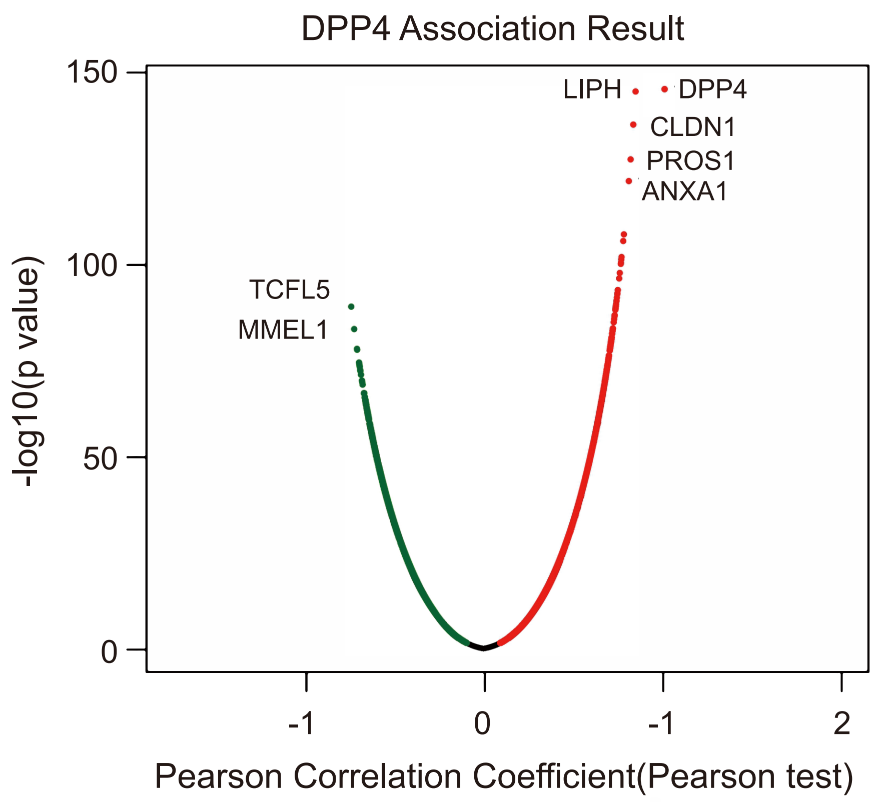
DPP4 Association Result
150
100
50
0
-log10(p value)
LIPH
DPP4
CLDN1
PROS1
ANXA1
TCFL5
MMEL1
-1
0
-1
2
Pearson Correlation Coefficient(Pearson test)

Supplementary figure 2. Co-expression network of DPP4.

Analysis via Linkedomics shows the co-expression network of DPP4. The red dots are genes positively related to the expression of DPP4 and the green dots negatively.

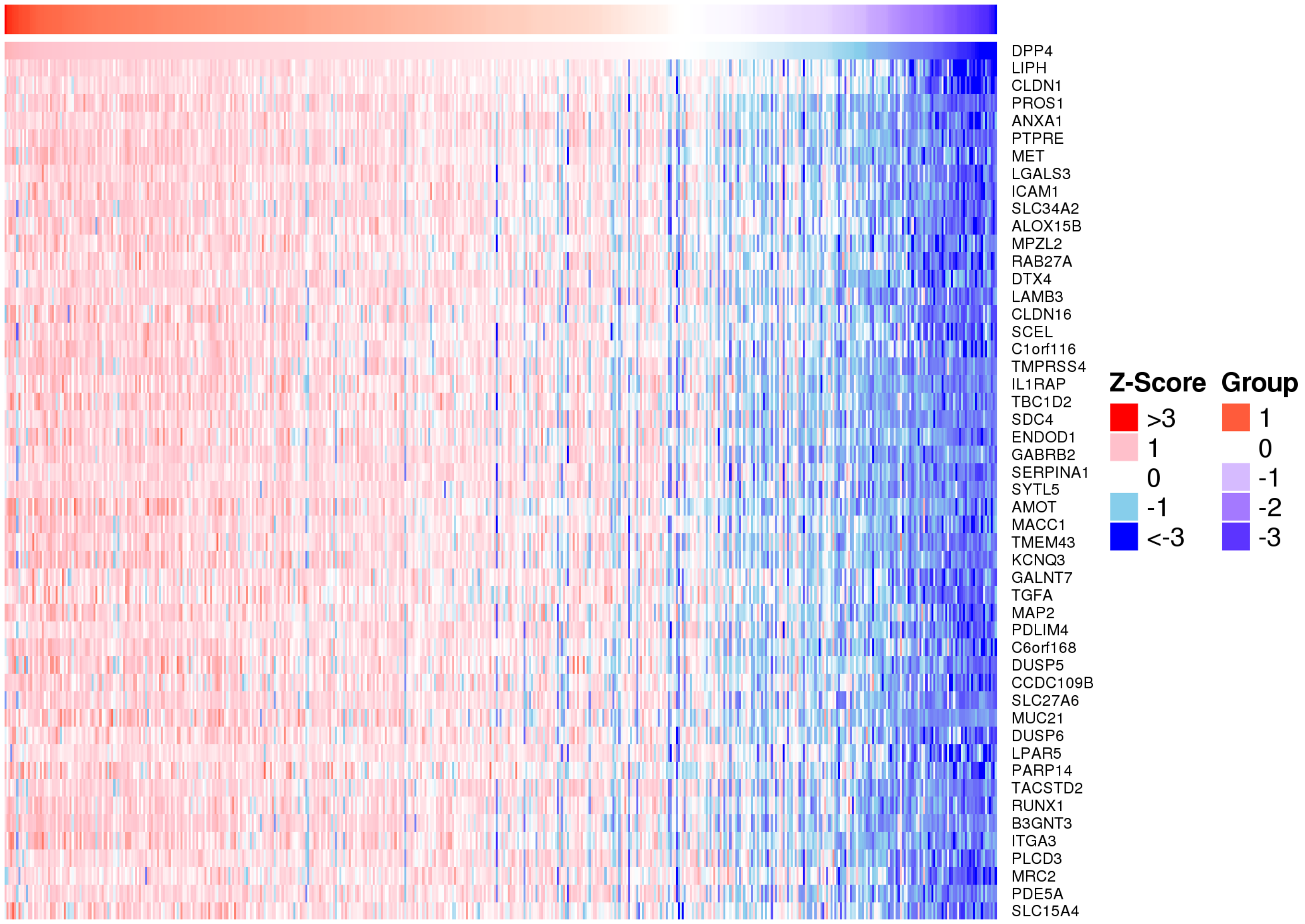
DPP4
LIPH
CLDN1
PROS1
ANXA1
PTPRE
MET
LGALS3
ICAM1
SLC34A2
ALOX15B
MPZL2
RAB27A
DTX4
LAMB3
CLDN16
SCEL
C1orf116
TMPRSS4
IL1RAP
TBC1D2
SDC4
ENDOD1
GABRB2
SERPINA1
SYTL5
AMOT
MACC1
TMEM43
KCNQ3
GALNT7
TGFA
MAP2
PDLIM4
C6orf168
DUSP5
CCDC109B
SLC27A6
MUC21
DUSP6
LPAR5
PARP14
TACSTD2
RUNX1
B3GNT3
ITGA3
PLCD3
MRC2
PDE5A
SLC15A4
Z-Score
>3
1
0
-1
<-3
Group
1
0
-1
-2
-3

Supplementary figure 3. Top 50 genes positively correlated with DPP4.

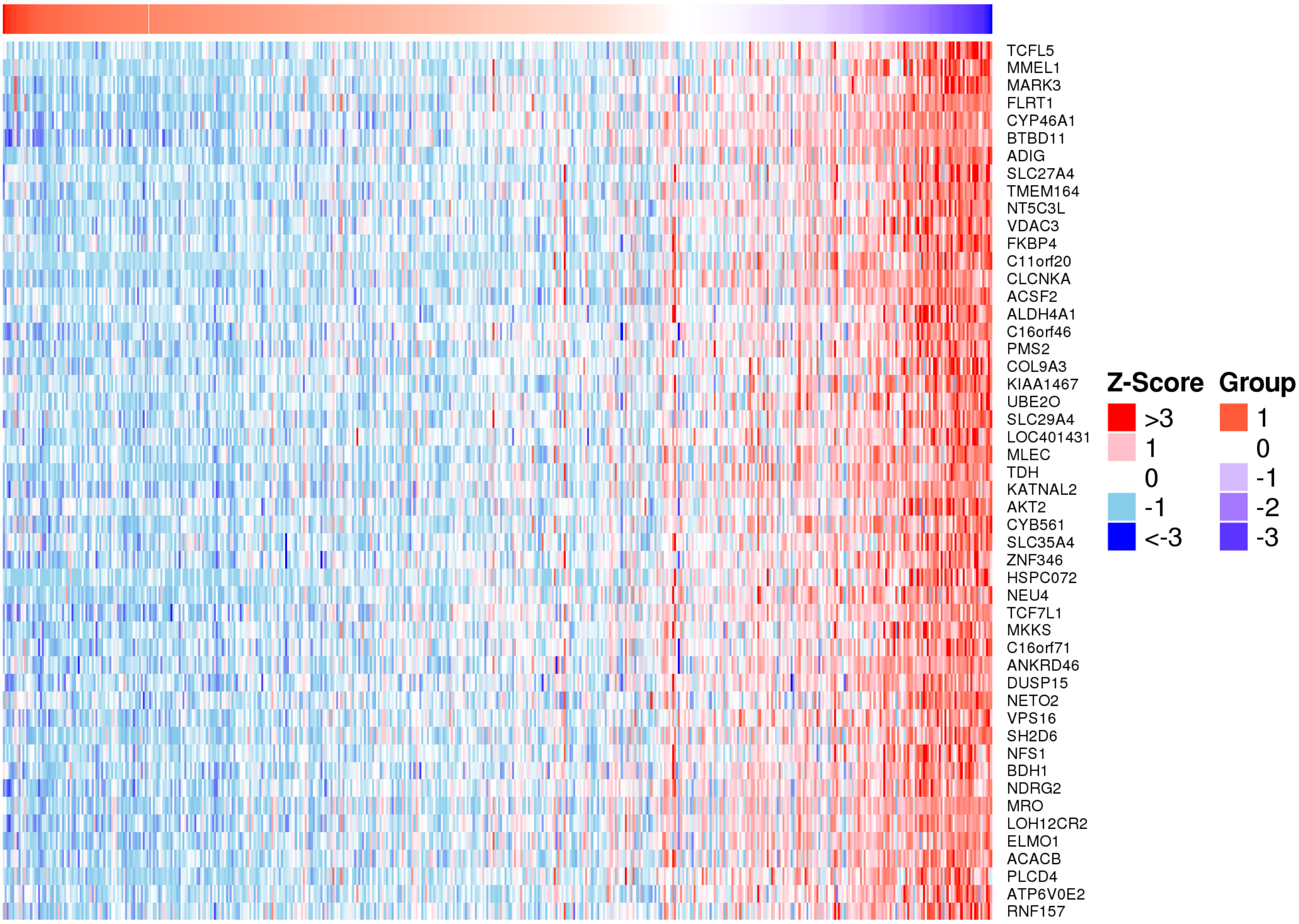
TCFL5
MMEL1
MARK3
FLRT1
CYP46A1
BTBD11
ADIG
SLC27A4
TMEM164
NT5C3L
VDAC3
FKBP4
C11orf20
CLCNKA
ACSF2
ALDH4A1
C16orf46
PMS2
COL9A3
KIAA1467
UBE2O
SLC29A4
LOC401431
MLEC
TDH
KATNAL2
AKT2
CYB561
SLC35A4
ZNF346
HSPC072
NEU4
TCF7L1
MKKS
C16orf71
ANKRD46
DUSP15
NETO2
VPS16
SH2D6
NFS1
BDH1
NDRG2
MRO
LOH12CR2
ELMO1
ACACB
PLCD4
ATP6V0E2
RNF157
Z-Score
>3
1
0
-1
<-3
Group
1
0
-1
-2
-3

Supplementary figure 4. Top 50 genes negatively correlated with DPP4.

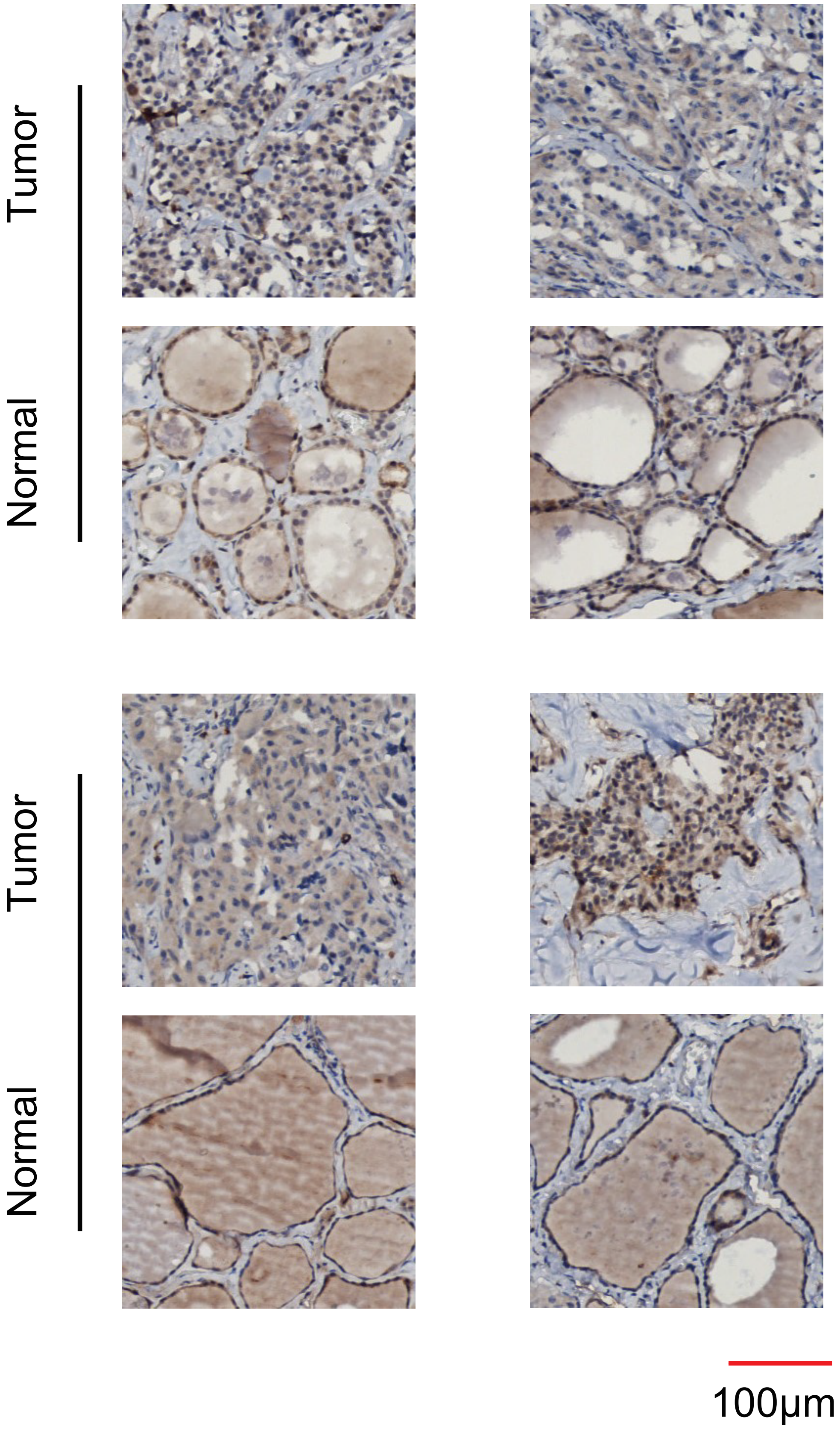
Tumor
Normal
Tumor
Normal
100μm

Supplementary figure 5. The IHC of additional 4 pairs of MTC and paracancerous tissues shows that the target protein DPP4 is mainly distributed in the cytoplasm of cancer cells and is stained brown by DAB. The thyroid follicular epithelium is slightly stained.
